# Supplementary material for: Resistance of melanoma to immune checkpoint inhibitors is overcome by targeting the sphingosine kinase-1
Source: Nat Commun. 2020 Jan 23;11:437. doi: 10.1038/s41467-019-14218-7 (PMC6978345; doi:10.1038/s41467-019-14218-7)
Supplement: Supplementary file 1 — Supplementary Information [file 41467_2019_14218_MOESM1_ESM.docx]

**Supplementary Information**

**Resistance of Melanoma to Immune Checkpoint Inhibitors is Overcome by Targeting the Sphingosine Kinase 1**

Imbert et al.,

**Supplementary methods**

**Establishment of shCtrl(2) or shSK1(3) Yumm cells.**

Yumm cells were transfected, with a SK1 shRNA (shSK1(3): GCTGCGGCTCTATTCTGTGCTCAGTCTGT) plasmid or a control non-targeting shRNA (shCtrl(2)) from Origene (#TF512690).

**CD4, CD8 and NK depletion experiments.**

Antibody depletions were performed by i.p. injections of 200 μg of control rat IgG (HRPN–Bio X Cell), anti-CD4 (GK1.5–Bio X Cell), anti-CD8β (53.5.8-Bio X Cell), anti-NK1.1 (PK136–Bio X Cell), on days -1, 0, 2, 8, 14 and 20 after tumor cell injection.

**Treg depletion**

C57BL/6^DTR/eGFP^ (DEREG) mice transgenic for the expression of diphtheria toxin receptor (DTR) and green fluorescent protein (GFP) under the influence of FoxP3 transcription factor gene were employed to deplete Treg and littermate wild type mice were used as control. DEREG mice have been kindly given by Dr. N. Fazilleau, INSERM U1043, Toulouse, France. Treg were depleted after tumor cell inoculation by i.p. injection of 1 μg diphtheria toxin (DT) at days 1, 4, 7 and 10 (Merck).

**SK1 rescue experiment**

Briefly, the murine Sphk1 cDNA from clone image (Life Science) was sub-cloned into the pCDNA3.1 plasmid (Thermofischer) that contains a neomycin cassette for selection. shSK1(3) Yumm cells were transfected either with an empty plasmid (Mock) or a plasmid encoding for the murine *Sphk1* cDNA (mSK1).

**Supplementary Figures.**


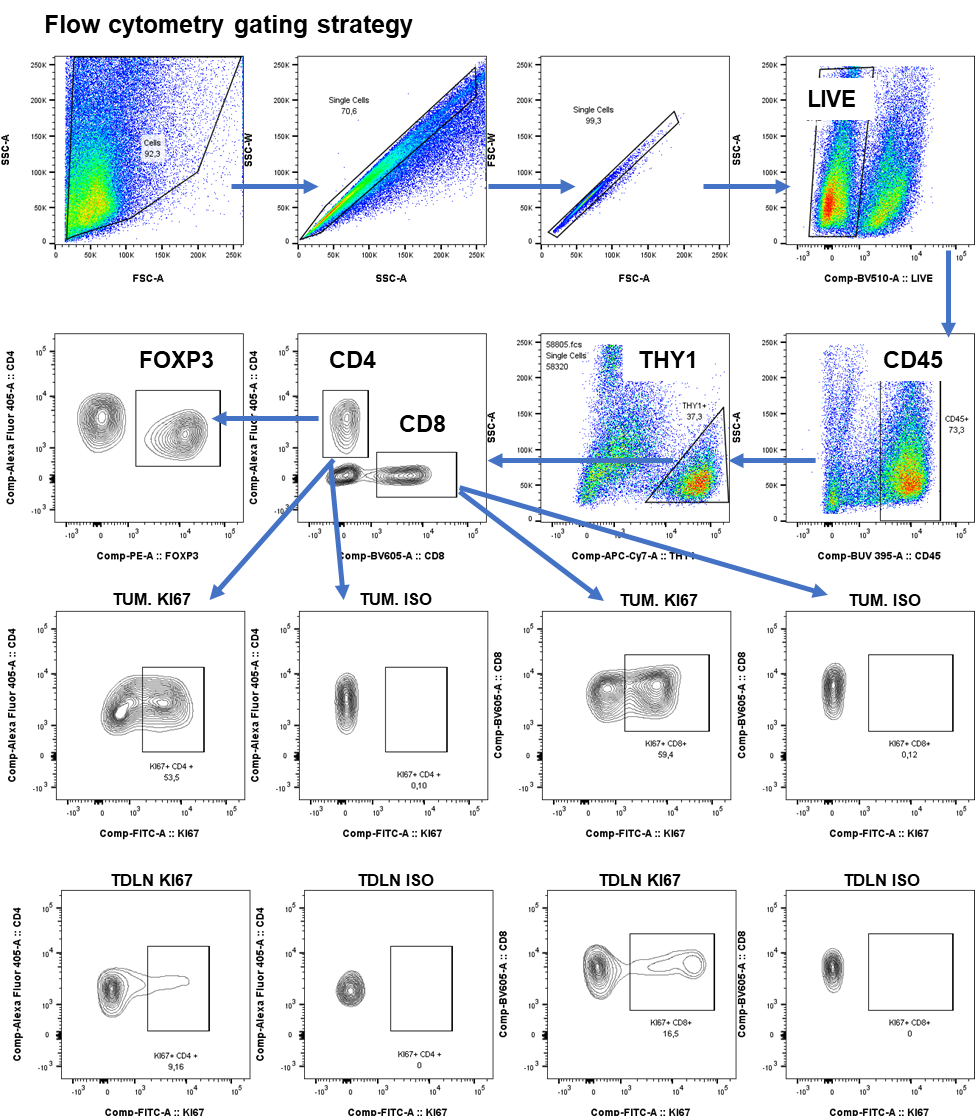


**Supplementary Figure 1. Representative flow cytometry gating strategy for T cells.** Flow cytometric analysis of KI67 positive CD4 and CD8 T cells in tumors (TUM) and tumor draining lymph node (TDLN). Iso: Isotype control

**
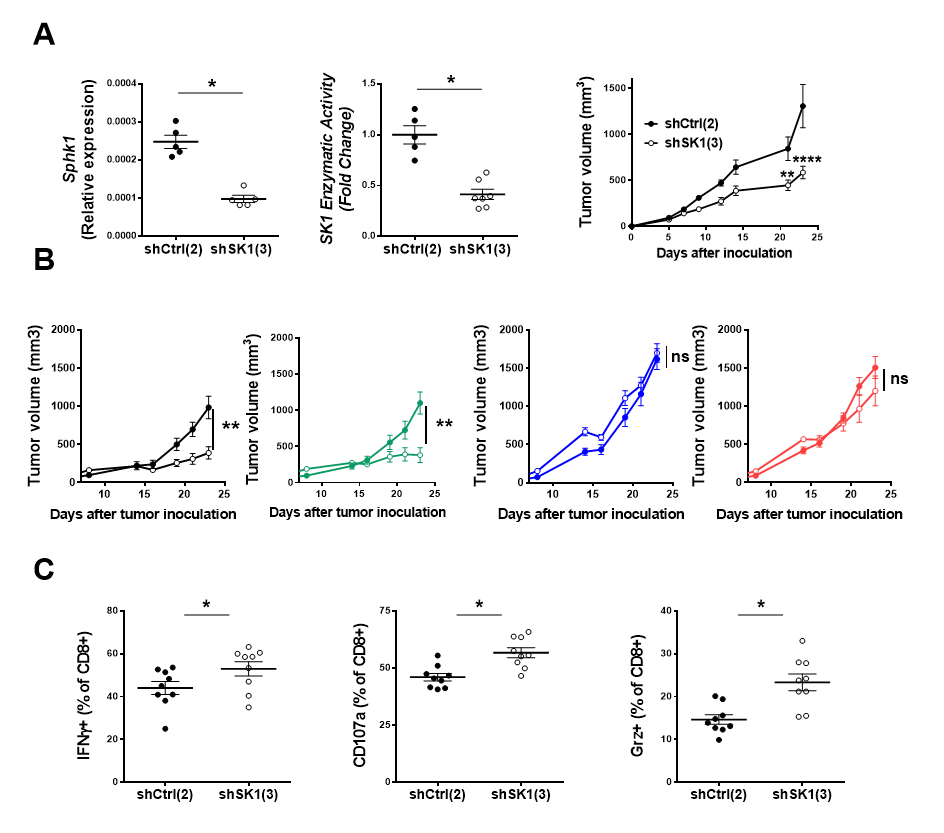
**

**Supplementary Figure 2.** SK1 downregulation enhances CD8 and CD4-dependent melanoma growth inhibition. (**A**) Relative *Sphk1* mRNA expression (left panel) and SK1 enzymatic activity (middle panel) were measured in Yumm cells transfected with a control shRNA (shCtrl(2); black columns/circles) or a SK1-targeted shRNA (shSK1(3); white columns/circles). shCtrl(2) (black points n=5) or shSK1(3) (white points n=7) Yumm cells were injected in C57BL/6 mice (right panel). (**B**) Tumor growth in mice depleted for CD4, CD8, or NK cells. Mice received an intraperitoneal injection of isotype antiboby (black lines) anti-CD4 (blue lines), anti-CD8 (red lines), and anti-NK1 (green lines) one day before intradermal injection of shCtrl(2) (full points) or shSK1(3) (empty points) Yumm cells, two days after and then every six days (n=7 mice/group). (**C**) Analyze of the antitumor activity of tumor-infiltrating CD8 T cells as measured by the expression of IFN-γ, LAMP-1 (CD107a) and granzyme B (Grz) at day 12 (n=9)

**Supplementary Figure 3. SK1 silencing increases CD8+/CD4+ Foxp3+ ratio in TdLN.** shCtrl and shSK1(1) Yumm cells were injected into the dermis of WT mice. The cell content of draining (TdLN) and non-draining (Non-TdLN) lymph nodes was analyzed by flow cytometry on day 11. Control shRNA (shCtrl; black points) or SK1-targeted shRNA (shSK1(1); white points) (n=9 mice/group). **(A)** Percentage of CD8+ (left panel), Foxp3+ CD4+ (Treg) (middle panel) T cells and CD8+/Treg ratio (right panel). **(B)** Percentage of CD8+ T cell (left panel) and Treg cells expressing Ki67 (middle panel) and CD8+ Ki67+/CD4+ Foxp3+ Ki67+ T cell ratio (right panel). Results are representative of at least 2 independent experiments (n = 9). Samples were compared using Kruskal-Wallis test with Dunn’s correction.

**Supplementary Figure 4. SK1 silencing reduces the expression of Treg-related molecules in melanoma tumors.** shCtrl or shSK1(2) Yumm cells were injected intradermally in C57BL/6 mice. At day 11, mice were sacrificed and tumors collected. Expression of *Foxp3, Tgfb1, Il10, Ccl17,* and *Ccl22* in shCtrl or shSK1(2) tumors was examined by RT-qPCR (n=5). Results are representative of 2 independent experiments. Samples were compared using Mann-Whitney test.

**Supplementary Figure 5. Impact of Foxp3+ Treg depletion on Yumm tumor growth.**

shCtrl or shSK1(1) Yumm cells were intradermally injected in DEREG or WT mice on day 0: (A) Mice were i.p. injected with 1µg of diphtheria toxin (DT) or PBS on days 1, 4, 7 and 10. (B) Analysis of Foxp3+ CD4+ T cells at day 8 in the tumor (TUM) and tumor-draining lymph nodes (TDLN) of DEREG mice injected with PBS or DT at days 1, 4 and 7. (C) Tumor growth of DEREG (empty green symbols with green lines) (n=12) or WT mice (full black symbols with black lines) (n=6). Tumor volumes are presented as means ± SEM. Samples were compared using a two-way ANOVA test.


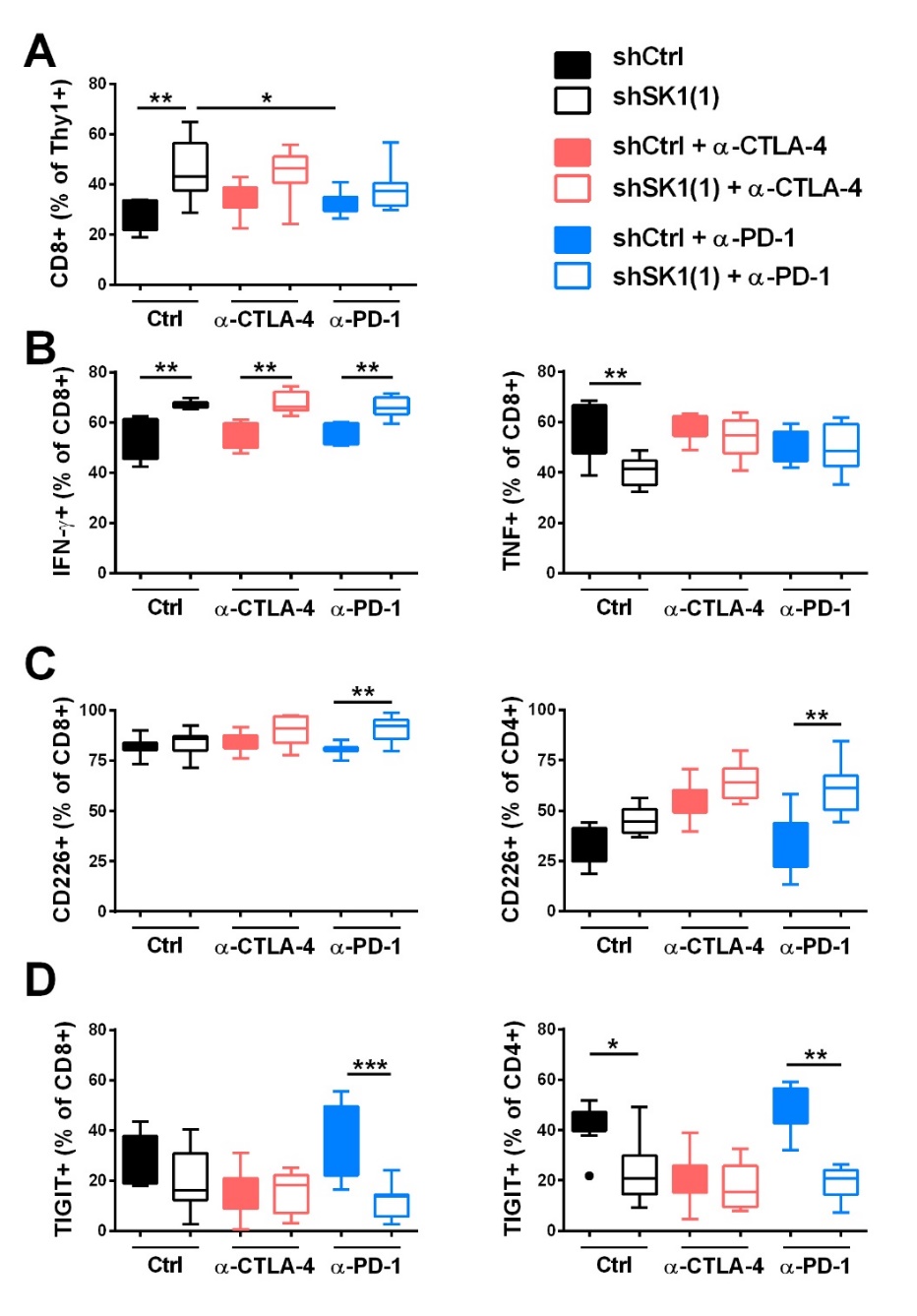


**Supplementary Figure 6. Characterization of CD4+ and CD8+ TILs upon ICI therapy.** Control shRNA (shCtrl, full boxes) or SK1-targeted shRNA (shSK1(1); empty boxes) Yumm cells were intradermally injected in C57BL/6 mice on day 0; Animals were then treated with control antibody (Ctrl; black boxes), anti-CTLA-4 (red boxes) or anti-PD-1 (blue boxes). TIL content was analyzed by flow cytometry on day 11 (n=10 mice/group). **(A)** Percentages of CD8+ TILs and CD8/Treg ratio. **(B)** Percentages of IFN-γ+ CD8+ and TNF+ CD8+ cells in tumors after PMA and Ionomycin stimulation. **(C)** Percentages of CD226+ CD8+ (left panel) or CD226+ CD4+ (right panel) T cells in tumors. **(D)** Percentages of TIGIT+ CD8+ (left panel) or TIGIT+CD4+ (right panel) T cells in tumors. Results are representative of 2 independent experiments. Samples were compared using Kruskal-Wallis test with Dunn’s correction.


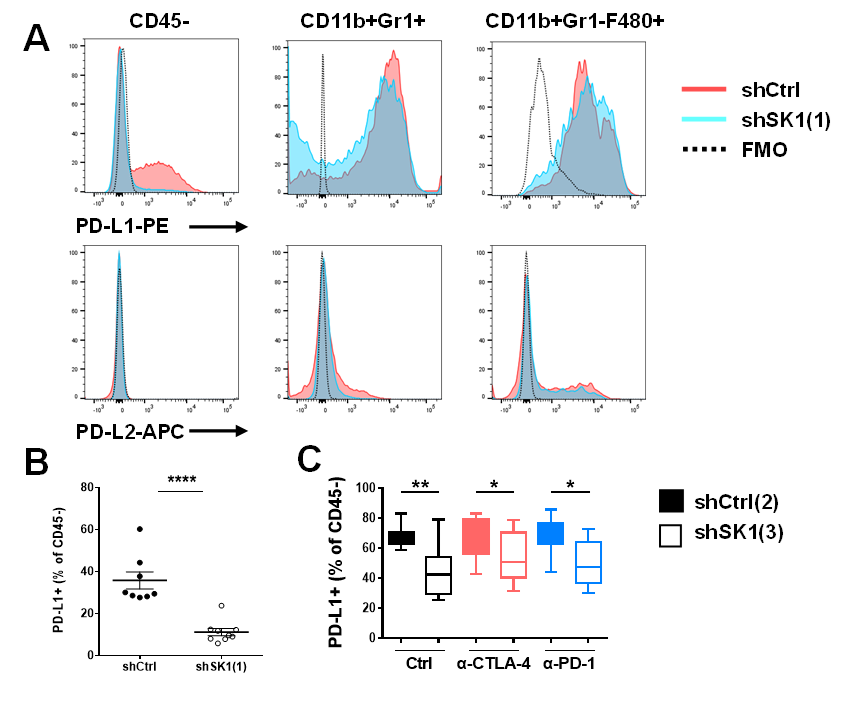


**Supplementary Figure 7. SK1 silencing decreases PD-L1 expression on CD45 negative cells in tumors.** (A). PD-L1 and PDL-2 expression on CD45-, CD11b+GR1+ (MDSC), CD11b+Gr1-F480+ (TAM) cells from tumors at day 11 after injection of control shRNA (shCtrl, red) or SK1-targeted shRNA (shSK1(1), blue) Yumm cells in C57BL/6 mice. The dotted line indicates the Fluorescence Minus One (FMO) control. (B). PD-L1 expression on CD45- cells from tumors at day 11 after injection of shCtrl (black points) or shSK1(1) (white points) Yumm cells (n=8). (C) PD-L1 expression on CD45- cells from tumors at day 11 after injection of shCtrl(2) (full boxes) or shSK1(3) (empty boxes) Yumm cells and treatment with isotype antibody (Ctrl; black boxes) or anti-CTLA-4 (red boxes) or anti-PD-1 (blue boxes) (n=8) (Samples were compared using Mann-Whitney test (B) or Kruskal-Wallis test with Dunn’s correction (C).


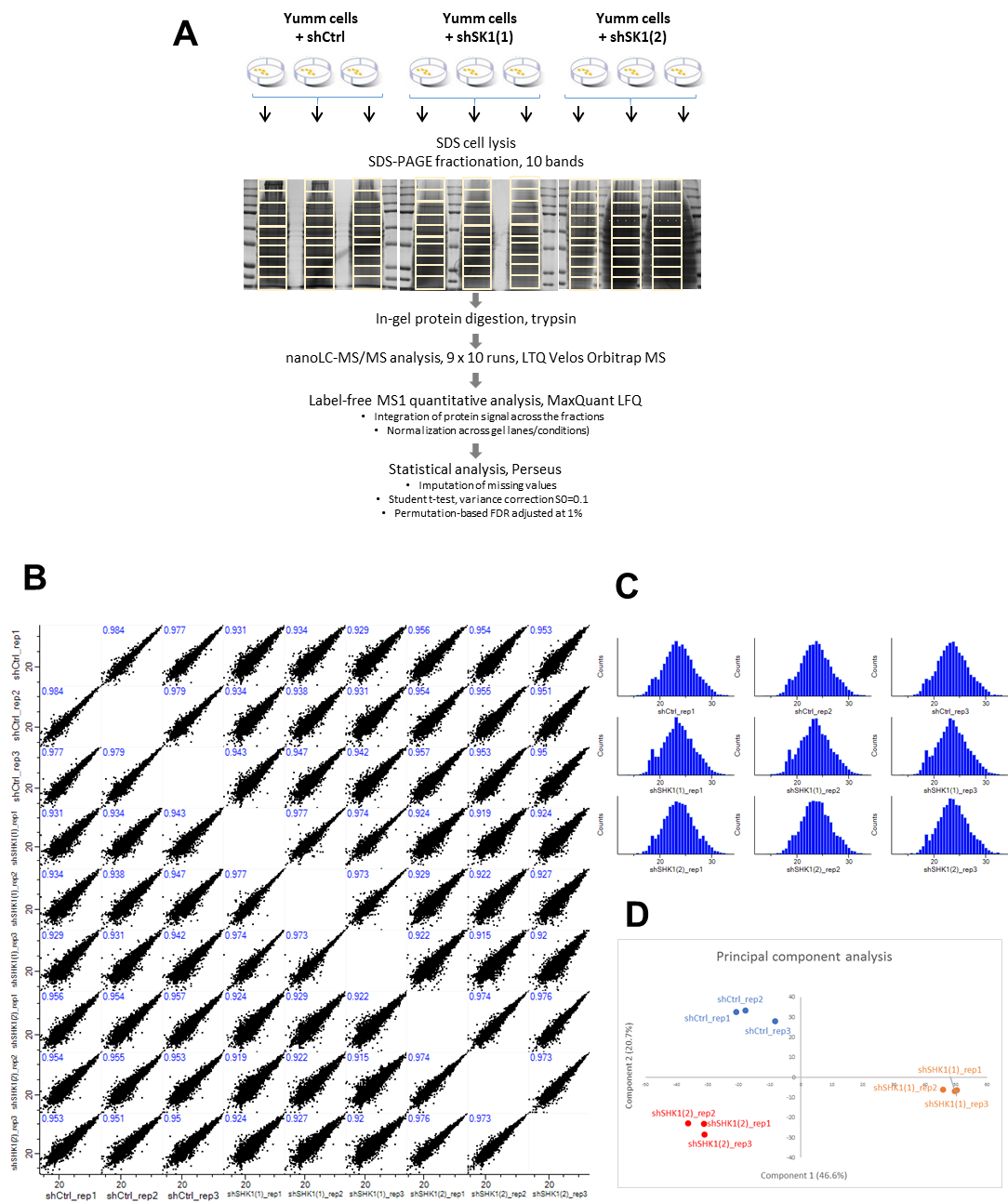


**Supplementary Figure 8.**  **Proteomic analysis: experimental design and quality control**. **(A)** Experimental design, sample preparation scheme and data processing procedure for the large-scale proteomic analysis. For each cell line (shCtrl, shSK1(1), shSK1(2)), 3 sample preparations were used and analyzed independently. **(B and C)** Quality control of the mass spectrometry data, showing repeatability between replicate experiments (denoted _rep1, _rep2, _rep3 for each condition) and global proteome stability between biological conditions (shCtrl, shSK1(1), shSK1(2)). **(B)** Multi scatter plots of LFQ intensity values for each protein after log2 transformation. Pearson correlation coefficients are indicated for each pairwise comparison. **(C)** Protein intensity distribution in each of the 9 analyzed samples. **(D)** Principal component analysis of the MS data, showing that little variance is introduced by the analytical process (_rep1, _rep2, _rep3), but essentially arises from the biological processes (silencing with shCtrl or sh targeting SK1).


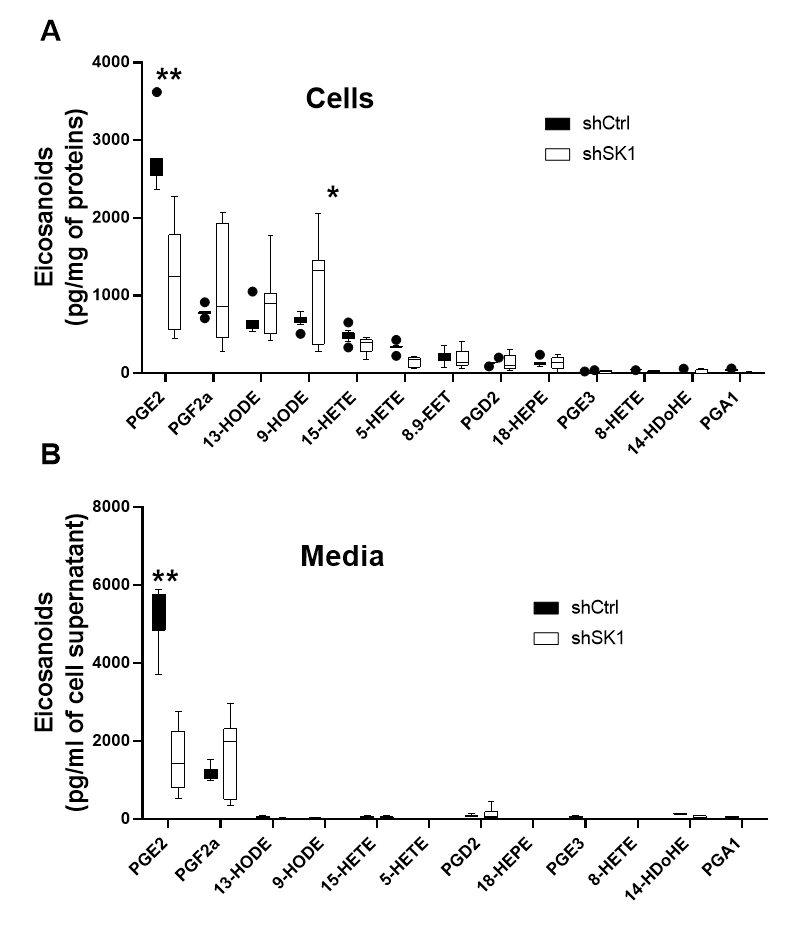


**Supplementary Figure 9. SK1 silencing specifically decreases PGE2 production.** Lipids were extracted from control shRNA (shCtrl; black columns) or SK1-targted shRNA (shSK1; white columns) cells **(A)** or culture medium **(B)** (n=9). Eicosanoid and related compounds were quantified by LC/MS. Concentrations of individual species were normalized to protein content **(A)** or to the volume of the medium **(B)**. The bottom-most and top-most horizontal lines, the lower and upper hinges, and the middle line of the boxplots indicate the minimum and maximum values, the 25th and 75th percentiles, and the median, respectively. Data are representative of 2 independent experiments. Data obtained with shSK1(1) and shSK1(2) were pooled. Samples were compared using two-way ANOVA test.

**
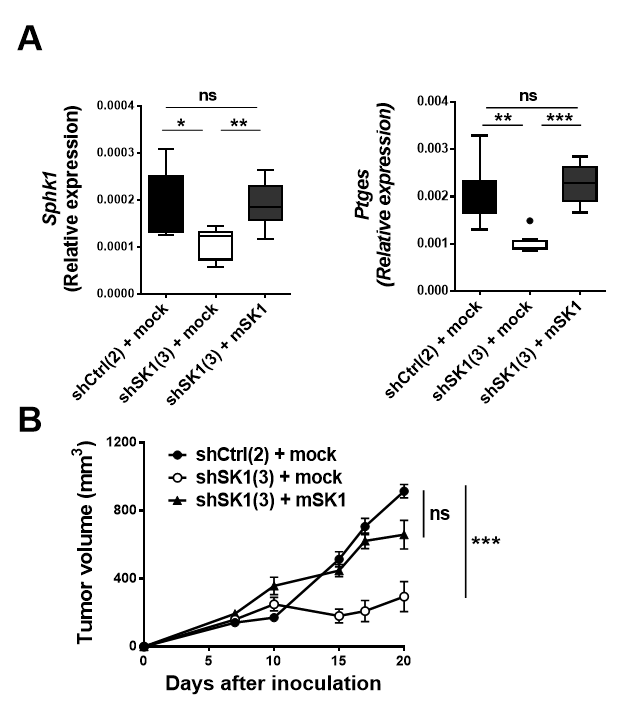
**

**Supplementary Figure 10. Re-expressing SK1 restores *Ptges* expression and tumor growth. (A)** *Sphk1* and *Ptges* relative levels of mRNA expression was measured in SK1-targeted shRNA shCtrl(2) ‘(black columns, n=8) or shSK1(3) Yumm cells transfected with an empty plasmid (mock; white columns n=9) or a plasmid encoding murine SK1 (mSK1; grey columns n=9). Data were compared using Kruskal-Wallis test with Dunn’s correction. The bottom-most and top-most horizontal lines, the lower and upper hinges, and the middle line of the boxplots indicate the minimum and maximum values, the 25th and 75th percentiles, and the median, respectively. (**B)** Mock-transfected shCtrl(2) (shCtrl(2) + mock; black circles n=10) or shSK1(3) (shSK1(3) + mock; white circles n=12) and mSK1-transfected (shSK1(3) + mSK1; black triangles n=9) shSK1(3) Yumm cells were injected in wild-type mice. Tumor volumes are means ± SEM. Samples were compared using two-way ANOVA test.
