# Supplementary material for: Resistance of melanoma to immune checkpoint inhibitors is overcome by targeting the sphingosine kinase-1
Source: Nat Commun. 2020 Jan 23;11:437. doi: 10.1038/s41467-019-14218-7 (PMC6978345; doi:10.1038/s41467-019-14218-7)
Supplement: Supplementary file 3 — Reporting Summary [file 41467_2019_14218_MOESM3_ESM.pdf]

nature research

Corresponding author(s): Céline Colacios

Last updated by author(s): Dec 3, 2019

Reporting Summary

Nature Research wishes to improve the reproducibility of the work that we publish. This form provides structure for consistency and transparency in reporting. For further information on Nature Research policies, see Authors & Referees and the Editorial Policy Checklist .

Statistics

For all statistical analyses, confirm that the following items are present in the figure legend, table legend, main text, or Methods section.

n/a	Confirmed	
☐	☒	The exact sample size (*n*) for each experimental group/condition, given as a discrete number and unit of measurement
☐	☒	A statement on whether measurements were taken from distinct samples or whether the same sample was measured repeatedly
☐	☒	The statistical test(s) used AND whether they are one- or two-sided<br>*Only common tests should be described solely by name; describe more complex techniques in the Methods section.*
☒	☐	A description of all covariates tested
☐	☒	A description of any assumptions or corrections, such as tests of normality and adjustment for multiple comparisons
☐	☒	A full description of the statistical parameters including central tendency (e.g. means) or other basic estimates (e.g. regression coefficient) AND variation (e.g. standard deviation) or associated estimates of uncertainty (e.g. confidence intervals)
☐	☒	For null hypothesis testing, the test statistic (e.g. *F*, *t*, *r*) with confidence intervals, effect sizes, degrees of freedom and *P* value noted<br>*Give P values as exact values whenever suitable.*
☒	☐	For Bayesian analysis, information on the choice of priors and Markov chain Monte Carlo settings
☒	☐	For hierarchical and complex designs, identification of the appropriate level for tests and full reporting of outcomes
☐	☒	Estimates of effect sizes (e.g. Cohen's *d*, Pearson's *r*), indicating how they were calculated

Our web collection on statistics for biologists contains articles on many of the points above.

Software and code

Policy information about availability of computer code

Data collection	BD FACSDiva v8.0.1; MaxQuant v1.5.3.30; Agilent 1290; G6460 triple Quad MS; Illumina HiSeq 2000 RNA
Data analysis	Graphpad Prism V6; STATA v13; Flow-jo software v10.5.3;

For manuscripts utilizing custom algorithms or software that are central to the research but not yet described in published literature, software must be made available to editors/reviewers. We strongly encourage code deposition in a community repository (e.g. GitHub). See the Nature Research guidelines for submitting code & software for further information.

Data

Policy information about availability of data

All manuscripts must include a data availability statement. This statement should provide the following information, where applicable:

- Accession codes, unique identifiers, or web links for publicly available datasets
- A list of figures that have associated raw data
- A description of any restrictions on data availability

https://xenabrowser.net (TCGA melanoma cohort) (Figure 7); www.oncomine.org (oncomine database) (Figure 1);
The mass spectrometry proteomics data have been deposited to the ProteomeXchange Consortium via the PRIDE partner repository with the dataset identifier PXD016535 (Figure 6).

Field-specific reporting

Please select the one below that is the best fit for your research. If you are not sure, read the appropriate sections before making your selection.

Life sciences study design

All studies must disclose on these points even when the disclosure is negative.

Sample size	No specific statistical test was used to predetermine the sample size.
Data exclusions	N/A
Replication	All experiments were representative of at least two independent experiments.
Randomization	No method of randomization was used.
Blinding	In vivo and in vitro experiments were monitored in a non-blinded fashion.

Reporting for specific materials, systems and methods

We require information from authors about some types of materials, experimental systems and methods used in many studies. Here, indicate whether each material, system or method listed is relevant to your study. If you are not sure if a list item applies to your research, read the appropriate section before selecting a response.

Materials & experimental systems

n/a	Involved in the study	
☐	☒	Antibodies
☐	☒	Eukaryotic cell lines
☒	☐	Palaeontology
☐	☒	Animals and other organisms
☒	☐	Human research participants
☐	☒	Clinical data

Methods

n/a	Involved in the study	
☒	☐	ChIP-seq
☐	☒	Flow cytometry
☒	☐	MRI-based neuroimaging

Antibodies

Antibodies used	All information are included in the methods.
Validation	Primary antibodies were validated by the manufacturer.

Eukaryotic cell lines

Policy information about cell lines

Cell line source(s)	All information are included in the methods.
Authentication	All information are included in the methods.
Mycoplasma contamination	Cell lines were routinely tested for the absence of mycoplasma contamination by PCR.
Commonly misidentified lines (See ICLAC register)	None

Animals and other organisms

Policy information about studies involving animals; ARRIVE guidelines recommended for reporting animal research

Laboratory animals	All information are included in the methods.
Wild animals	None
Field-collected samples	Tumours, lymph nodes, lungs
Ethics oversight	All experimental protocols were approved by the local ethic committee (Midi-Pyrénées, France) and are in compliance with the French and European regulations on care and protection of laboratory animals.

Note that full information on the approval of the study protocol must also be provided in the manuscript.

Clinical data

Policy information about clinical studies

All manuscripts should comply with the ICMJE guidelines for publication of clinical research and a completed CONSORT checklist must be included with all submissions.

Clinical trial registration	None
Study protocol	N/A; tumour samples were collected retrospectively.
Data collection	Tumour samples were collected retrospectively.
Outcomes	N/A

Flow Cytometry

Plots

Confirm that:

- [x] The axis labels state the marker and fluorochrome used (e.g. CD4-FITC).
- [x] The axis scales are clearly visible. Include numbers along axes only for bottom left plot of group (a 'group' is an analysis of identical markers).
- [x] All plots are contour plots with outliers or pseudocolor plots.
- [x] A numerical value for number of cells or percentage (with statistics) is provided.

Methodology

Sample preparation	All information are included in the methods.
Instrument	BD LSR Fortessa X-20
Software	BD FACSDiva v8.0.1
Cell population abundance	N/A
Gating strategy	See supplementary Figure 2.

- [x] Tick this box to confirm that a figure exemplifying the gating strategy is provided in the Supplementary Information.
